# Mental status assessment of disaster relief personnel by vocal affect display based on voice emotion recognition

**DOI:** 10.1186/s40696-017-0032-0

**Published:** 2017-04-08

**Authors:** Shunji Mitsuyoshi, Mitsuteru Nakamura, Yasuhiro Omiya, Shuji Shinohara, Naoki Hagiwara, Shinichi Tokuno

**Affiliations:** 1 Verbal Analysis of Pathophysiology, Room 208 Molecular and Life Innovation Building, The University of Tokyo Hospital, 7-3-1 Hongo, Bunkyo-ku, Tokyo, 113-8655 Japan; 2 PST Inc., Yokohama, Japan

**Keywords:** Disaster relief, Stress assessment, Noninvasiveness, Screening, Vocal affect display, Voice emotion analysis

## Abstract

**Background:**

Disaster relief personnel tend to be exposed to excessive stress, which can be a cause of mental disorders. To prevent from mental disorders, frequent assessment of mental status is important. This pilot study aimed to examine feasibility of stress assessment using vocal affect display (VAD) indices as calculated by our proposed algorithms in a situation of comparison between different durations of stay in stricken area as disaster relief operation, which is an environment highly likely to induce stress.

**Methods:**

We used Sensibility Technology (ST) software to analyze VAD from voices of participants exposed to extreme stress for either long or short durations, and we proposed algorithms for indices of low VAD (VAD-L), high VAD (VAD-H), and VAD ratio (VAD-R), calculated from the intensity of emotions as measured by voice emotion analysis. As a preliminary validation, 12 members of Japan Self-Defense Forces dispatched overseas for long (3 months or more) or short (about a week) durations were asked to record their voices saying 11 phrases repeatedly across 6 days during their dispatch.

**Results:**

In the validation, the two groups showed an inverse relationship in VAD-L and VAD-H, in that long durations in disaster zones resulted in higher values of both VAD-L and VAD-R, and lower values of VAD-H, compared with short durations. Interestingly, phrases produced varied results in terms of group differences and VAD indices, demonstrating the sensitivity of the ST.

**Conclusions:**

A comparison of the values obtained for the different groups of subjects clarified that there were tendencies of the VAD-L, VAD-H, and VAD-R indices observed for each group of participants. The results suggest the possibility of using ST software in the measurement of affective aspects related to mental health from vocal behavior.

## Background

It is known that disaster relief personnel experience excessive stress, which can cause mental disorders such as post-traumatic stress disorder or depression [1–4]. To prevent personnel from mental disorders, frequent assessment of mental status is important. There may be many candidates of indices for assessing magnitude of stress on subjects, however, the indices for frequent assessment must be noninvasive and reasonable ones considering burden on subjects.

Human emotion is influenced by external circumstances such as extreme stress [5] in addition to changing spontaneously as a result of internal activities. Emotion varies widely between individuals and situations because it involves numerous elements and factors. Though there is wide variation in emotion, in general the influence of external circumstances can be observed as a difference between groups that are distinguished by their respective environmental conditions. Emotions can be assessed in non-contact ways, as humans can usually identify the emotions of other individuals without requiring contact, much less the need for invasive techniques. Measurement of other biomarkers, in most cases, requires contact with the body or invasion into the body. Also many other biomarkers require expendable supplies for measurement. This causes relative high costs of measurements.

Methods for measuring people’s emotions include approaches that use various brain function measurement techniques to study activity in areas of the brain thought to be linked to emotions [6], but these commonly require large-scale and costly equipment and are difficult to use on a daily basis.

Emotion-measuring methods that can be routinely employed and are low in cost include techniques that distinguish between facial expressions [7] and techniques that analyze the voice [8–10]. Recognition of facial expressions in a given individual is relatively easy, but between individuals, bias due to individual variation is a problem, and it is difficult to turn this into a generic technique without customization. There are also cultural biases of facial expression [11]. An attempt to detect stress from facial affect display [12] has been reported. In that study [12], two emotional expressions, those of anger and disgust, were considered equivalent as markers for the existence of stress, and other measurements such as via questionnaires and biomarkers were not carried out.

Bias due to individual variation also exists in vocal emotion analysis, but in Sensibility Technology (ST) [8–10], which is one of these techniques, this bias is addressed by collecting voice samples from a large number of subjects. Furthermore, ST does not depend on the linguistic content of utterances and therefore promises to be usable in a wide range of applications.

If the precision of voice emotion recognition by ST corresponds to or is higher than that of a subject’s subjective assessment, we hypothesized that it should be possible to detect changes arising from the pathology of an affective disorder with an accuracy level the same as or higher than that of a self-administered questionnaire.

Vocal affect displays (VADs) are characteristics of vocal features derived through voice emotion analysis. As markers for the overall emotional state, they can reflect changes brought on by environmental influences. For the present study, we devised algorithms for deriving measures of overall low VAD (VAD-L) indicating negative emotional states, high VAD (VAD-H) indicating positive emotional states, and VAD ratio (VAD-R) based on emotions (anger, joyfulness, calmness, sorrow, excitement) that have been recognized from the voice using ST. In this preliminary experiment to examine feasibility of assessment using the measures derived with this algorithm, voice data were collected from members of the Japan Self-Defense Forces who were deployed in a disaster relief operation after the Haiti earthquake, and then the relationship between the period of deployment and the VAD indices derived from the voices was studied.

## Methods

### Voice emotion analysis system

We used the software Sensibility Technology (ST) Ver. 3.0 (AGI Inc., Tokyo, Japan) [8–10] as a voice emotion analysis system for determining emotions from subjects’ voices.

The categories of emotional elements detected by the ST software are “anger,” “joy,” “sorrow,” “calmness,” and “excitement.” Output values include the amplitude of each emotion as detected in the input voice, in the form of integer values from 0 to 10. A value of 0 means that the input voice does not include the emotion at all; a value of 10 means that the input voice includes the emotion to a very high degree.

The ST software was developed based on the idea that excitement is different from the other four emotions because it contains arousal, which is related to other emotional elements. Therefore, evaluation of the sample voices and construction of the classifier for degree of excitement were independent from those for the other four emotions [8]. The ST software outputs the percentages of each of the other four emotions out of the total of all four categories; so, for example, when joyfulness declines, the percentages of the other three emotions increase accordingly. We treat degree of excitement as the primary indicator of VAD.

The minimum unit of vocal expression analyzed by the ST software is an “utterance,” which means a continuous vocalization between two breaths. In practice, the start of an utterance is detected when it changes from the silent state to the uttering state and the uttering state continues for a certain duration, and the end of an utterance is detected when it changes from the uttering state to the silent state for a certain duration. Whether the state is silent or uttering is determined by comparison with a specified threshold. In this study, the threshold was adjusted manually for each recording because the volume of voices was affected by the subjects and by the recording conditions.

### Algorithms for vocal affect display indices

The outputs produced by the ST software indicate the intensity of the emotional elements; they are not a direct expression of the overall emotional state. We defined three indices to express the overall emotional state of human emotion detected from one’s voice, called vocal affect displays (VADs). The intent in creating the VAD indices is for use in screening for subjects who are not in a positive emotional state. The algorithms for calculating VAD indices were based on our own heuristics, such as observations of ours based on medical interviews and medical guidelines. Two of the three indices are for the quantification of low VAD (VAD-L) and high VAD (VAD-H). The third index is the VAD ratio (VAD-R), intended for use in screening.

**Fig. 1:**
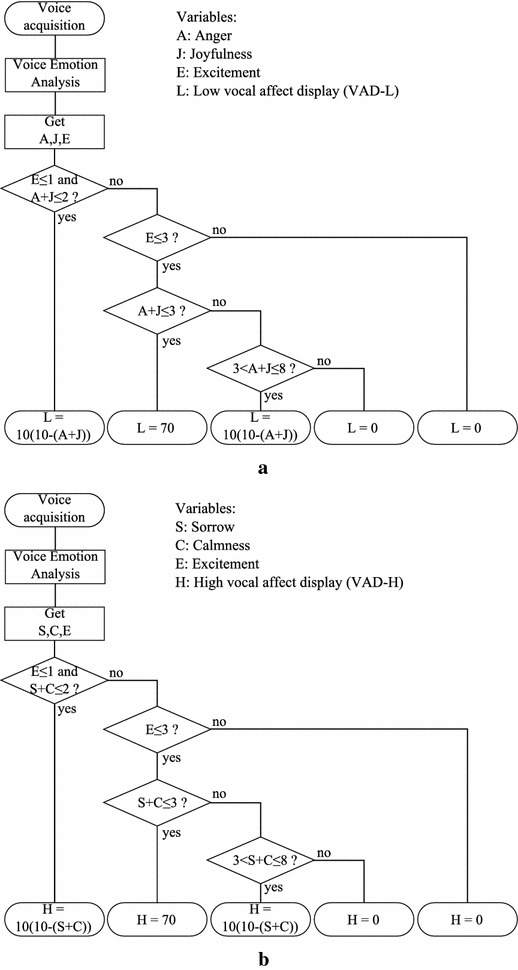
Flow charts of algorithms for calculating vocal affect display (VAD) indices. The ST software is used as the Voice Emotion Analysis module. **a** Determination of low VAD index (VAD-L). **b** Determination of high VAD index (VAD-H)

Details of the algorithms are shown in Fig. 1 in two flow charts. The input variables are the outputs from the voice emotion analysis obtained from ST and are represented as follows:

- Anger: A
- Joyfulness: J
- Calmness: C
- Sorrow: S
- Degree of Excitement: E

Their values are whole numbers between 0 and 10, inclusive.

The two Vocal Affect Display (VAD) index values output by the algorithms are represented as follows:

- Low VAD (VAD-L): L
- High VAD (VAD-H): H

The first algorithm calculates an index that reflects the extent to which emotions that are considered strongly related to low emotion exercise overall control; the second algorithm does the same for high emotion. Both algorithms focus first on the degree of excitement (E) found by ST, and then judge the constitution of total emotions by focusing on differences in the intensities of specific emotion categories. The difference between the two algorithms is the set of emotions used in the latter portion of the process.

These algorithms were constructed based on the fact that the degree of excitement as measured by ST (mainly measured by an acoustic parameter related to the basic frequency) reflects the degree of activity of that part of the brain that controls emotions, indicating the intensity of the overall emotions [8]. A major characteristic of the pathology of an emotional disorder is a loss of emotion, so the strength of emotion in the brain is estimated based on degree of excitement in order to capture this characteristic.

We hypothesized that when the degree of excitement E is 3 or less on a scale of 1–10, emotions are almost as torpid as in a low-emotion state; and when it is less than 1, emotions are markedly reduced or lost. Based on this, the upper threshold of E for the low-emotion state was set to 3, and the upper threshold of E for the most serious state was set to 1. These thresholds were chosen with reference to the deviation of voice characteristics (a lack in energy such as vibrato, or near inaudibility) heard by one of the authors when observing clinical psychologists or physicians questioning depression patients since 2002.

**Fig. 2:**
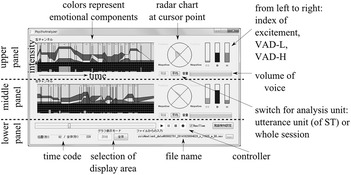
User interface. The *upper panel* is for the *left channel* of stereo sound data, and the *middle panel* is for the *right channel*; the components of the *upper panel* and the *middle panel* are same. The *lower panel* is for control and settings

We built an interface incorporating these two algorithms that visualizes short-term changes across utterance units and long-term tendencies by averaging over a session; this is depicted in Fig. 2.

For a given session, VAD-R is defined as the ratio of the session average of VAD-L to the session average of VAD-H.

### Validation of algorithms

#### Participants

We recruited 12 members of the Japan Self-Defense Forces who were dispatched to Haiti to provide assistance after the earthquake of January 12, 2010, as subjects. They were all male, native Japanese speakers between 30 and 59 years of age. The members gave their oral consent to the recording of their voices. The protocol was approved by the Ethics Committee of the National Defense Medical College (No. 624).

The test subjects were divided into two groups—those who stayed in Haiti for long periods (3 months or more), labeled as Group L, and those who stayed there for short periods (about 1 week), labeled as Group S—and their voices were recorded during time periods when Group S subjects were in Haiti. Subjects in Group L had been in Haiti for more than a month before the acquisition of their voices. Voice acquisitions were carried out twice, in July and December of 2010. Each time, 3 subjects in Group L and 3 subjects in Group S participated. No subject participated in both July and December.

#### Voice acquisition

The subjects’ voices were recorded using the voice recorder ICR PS502RM (Sanyo Electric, Osaka, Japan). The recording format was linear PCM, the sampling frequency was 44.1 kHz, the quantized bit number was 16 bits, the recording level was low, and the directivity switching was “Zoom.” The microphone auto level control, low cut filter, recording peak limiter, voice-activated system (VAS) setting, and automatic silence split were all off.

The voices were collected by having the subjects read fixed phrases (excluding one phrase or word depending on how the participants were feeling) twice a day: every morning and every night for six consecutive days. The phrases they read are shown in Table 1.

**Table 1:** Manuscript of phrases read by subjects

No.	Phrase	Translation
1	I-ro-ha-ni-ho-he-to	(No means like “a-b-c”)
2	I-ro-ha-ni-ho-he-to	(No means like “a-b-c”, repeat)
3	Watashi ha jieikan de, nihon kara kiteimasu	I belong to the Self-Defense Force and come from Japan
4	Tsukarete guttari shiteimasu	I am tired and am dead tired
5	Totemo genki desu	I am very cheerful
6	Kinou ha yoku nemuremashita	I was able to sleep well yesterday
7	Syokuyoku ga arimasu	I have an appetite
8	Okorippoi desu	I am irritable
9	Huan de ippaidesu	I am in great anxiety
10	Kokoro odayaka desu	My heart is calm
11	Kyono kibun wo iro de arawasuto {color} desu	I am {color} when I express today’s feeling with a color

All phrases were in Japanese, and all subjects were native Japanese speakers

Each test subject was instructed to operate his own voice recorder while holding it with the microphone toward his mouth at a distance of about 15 cm from his mouth.

#### Voice emotion analysis

The recorded voices were analyzed after all the test subjects had returned to Japan. All voices were transferred as electronic files from the voice recorders to a computer with the ST software. When the recorded voices were checked, it was discovered that the voice recording level for 2 long-stay subjects and 1 short-stay subject were so high that the sounds were clipping, so these recorded voices were discarded because they were considered unsuitable for analysis.

#### Analysis

A read-through of all of the fixed phrases was defined as one session. Each series of utterances was divided into utterance units, each of which can be uttered with one breath. Each session included multiple ST utterance units. The emotion in the background in each utterance unit was analyzed based on the utterance voices. Algorithms to judge how closely the total hypothesized emotion (based on the combination of emotions detected from the utterance units) matched a low-emotion state were proposed.

The averages of a subject’s respective VAD indices for the utterance units in a session were defined as that subject’s VAD indices for that session. We used one-way analysis of variance to examine whether there was a significant difference between the distributions of VAD indices acquired from the two subject groups, Group S and Group L. The assessment was done using Microsoft Excel 2010 test functions.

## Results

Table 2 shows the results of one-way analyses of variance comparing the VAD indices of voices acquired from Group S subjects (n = 5) with those of voices acquired from Group L subjects (n = 4).

**Table 2:** Results of one-way analysis of variance comparing VAD indices calculated for two subject groups

	Sum of squares	Degrees of freedom	Mean square	*F*-ratio	Significance level	η^2^
*(1) VAD-L*						
Between groups	1654	1	1654	1.213	0.3071	0.1477
Within groups	9545	7	1364			
Total	11,199	8				
*(2) VAD-H*						
Between groups	558.0	1	558.0	1.756	0.2267	0.2006
Within groups	2224	7	317.7			
Total	2782	8				
*(3) VAD-R*						
Between groups	204.4	1	204.4	3.096	0.1219	0.3066
Within groups	462.2	7	66.03			
Total	666.6	8				

**Fig. 3:**
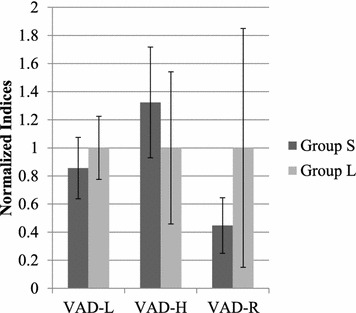
Comparison of normalized VAD indices by session between subject groups. Group S consisted of subjects with short duration of stay in disaster area, and Group L consisted of subjects with long duration of stay in disaster area. Indices were normalized so that the averages for Group L became 1 in each index. Heights of *bars* show the means of the VAD indices by session for each subject group, and *error bars* show the standard deviations

Compared with sessions of Group S subjects, there are tendencies that sessions of Group L subjects had higher VAD-L (average 67.58, standard deviation 15.14 for Group L; average 57.75, standard deviation 14.67 for Group S) and VAD-R (average 6.189, standard deviation 5.261 for Group L; average 2.770, standard deviation 1.224 for Group S) values and lower VAD-H (average 17.49, standard deviation 9.426 for Group L, average 23.13, standard deviation 6.892 for Group S) values; however, these indices did not show significant differences between Group S and Group L by analysis of variance (significance levels are 0.3071, 0.2267 and 0.1219 each for VAD-L, VAD-H and VAD-R). VAD-R showed the largest F-ratio among VAD-L (1.213), VAD-H (1.756), and VAD-R (3.096). On the other hand, effect sizes η^2^ of VAD indices are 0.1477, 0.2006 and 0.3066 each for VAD-L, VAD-H and VAD-R. Figure 3 shows the normalized means and standard deviations of VAD-L, VAD-H, and VAD-R for each subject group.

**Fig. 4:**
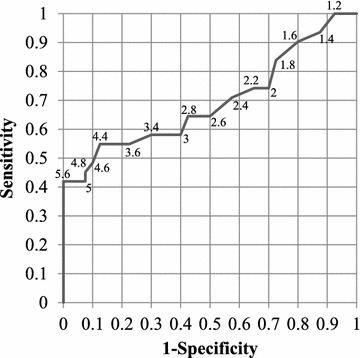
ROC curve of VAD-R index as a binary classifier. This curve shows performance of VAD-R index when the index is used to distinguish whether the sample is based on long-stay subjects or on short-stay subjects. Numbers on the trace are the threshold values of VAD-R to discriminate sessions by long-stay subjects

The receiver–operating characteristic (ROC) curve for a case where the VAD-R index is considered to be a classifier for duration of stay is shown in Fig. 4. The area under this ROC curve is 0.695.

**Fig. 5:**
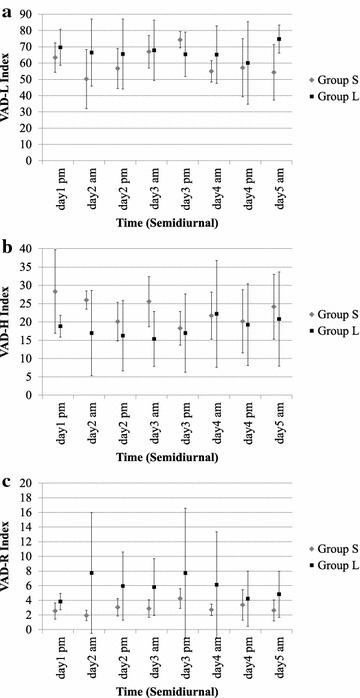
Change in VAD indices over time. The start of the recording test was defined as the starting point of this graph (day 1). *Markers* show means of VAD indices by time for each subject group, and *error bars* show standard deviations. **a** VAD-L, **b** VAD-H, **c** VAD-R

Figure 5 shows the results of grouping and averaging the data according to the relative passage of time during the measurement period (half-day units) in order to calculate the VAD-L, VAD-H, and VAD-R indices, and plotting their change over time. No significant difference was found between Group S and Group L at a significance level of 0.05.

**Fig. 6:**
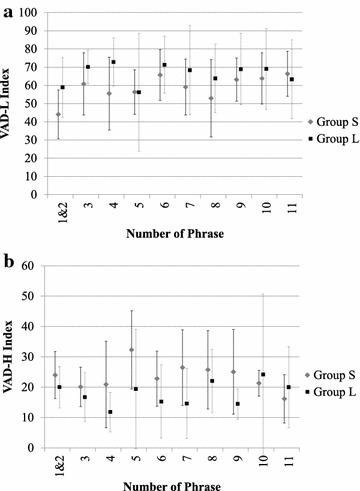
Relationship between read phrases and VAD indices. *Markers* show means of VAD indices by phrase for each subject group, and *error bars* show standard deviations. **a** VAD-L, **b** VAD-H

Figure 6 shows the averaged VAD-L and VAD-H indices for the data when grouped by the phrases that were read out. (VAD-R cannot be calculated because VAD-R is defined per session.) The second phrase is a repeat of the first, so they are combined on the graphs. No significant difference was found between Group S and Group L at a significance level of 0.05.

## Discussion

The indices we have proposed are intended to indicate some of the differences in VAD between two groups of subjects, which are those who had a long stay (of 3 months or more) (Group L) and those who had a short stay (of about a week) (Group S) in a disaster area. Subjects of Group L had tendencies to show high VAD-L and VAD-Rand low VAD-H. However, differences in the VAD indices between Group L and Group S were not significant; the effect size η^2^ of VAD-R was 0.3066.

The results of recognizing and analyzing emotions from the voices of the same test subjects [13] revealed qualitative differences in the patterns of emotional expression between the long-stay test subjects and the short-stay test subjects, but it is difficult to significantly categorize the two groups using only one emotional indicator. Looking at trends in changes over time, as shown in Fig. 5, we see that changes in the VAD-L index are nearly the reverse of trends in the change in joyfulness or degree of excitement in emotions that was previously observed [13], and when we compare the values for the short-stay subjects with those for the long-stay subjects, we see that the only reversal occurs in the afternoon of the third day. An analysis by phrase, as shown in Fig. 6, shows a tendency for the VAD-L index in particular to be nearly the reverse of trends in the change in degree of excitement [13], so we can infer that the effect of excitement is important.

On the other hand, an examination of the ROC curve and the values of area under the curve (AUC) indicate that the sensitivity and degree of uniqueness are insufficient to set specific threshold values for the VAD-R index and judge whether or not a sample is from a long-stay subject using the threshold values. However, all sessions showing a VAD-R index greater than 5.6 were acquired from participants of Group L, therefore specificity is sufficient in high VAD-R regions.

Being sent to a disaster area is itself the cause of stress on the personnel sent, and presumably, the longer the test subjects stay, the greater the stress they are subjected to. On the other hand, there were possibilities that the participants of Group L acclimated themselves to the environment, and the participants of Group S were stressed from the acute change of environment. In this study, it is presumed from the tendencies of VAD indices that accumulated stress was more dominant. However, further experiments including measurements of pre-dispatch and post-dispatch terms will be required to evaluate temporal changes such as acute and chronic effects. In addition, comparison with non-dispatched population will help make effects of a dispatch clear.

It is further assumed that there are large individual differences in the ability to withstand stress and that the environments the dispatched personnel actually experience are not necessarily uniform, so presumably there are also cases where distinguishing a group at the individual level based on a threshold value will not necessarily be effective. In addition, the participants in this study were all male, because we had no chance to recruit female participants. It is also a future issue to examine whether any sexual difference exists or not and evaluate feasibility of stress assessment using voice for female participants.

The test subjects who participated in this study were sent overseas to assist at an earthquake scene, so it was impossible to hypothesize about their psychological condition based on biomarker information other than voice or on questionnaires. Therefore, the information that can be compared with the voices is limited to the length of time they were dispatched. As a future issue, validity of the proposed method must be evaluated by using conventional methods.

Additionally we used reading of fixed phrases to be recorded. It is presumed that emotions are less expressed when someone read written phrases in comparison to spontaneous speech, like the case of detection of depression from speech [14]. In this study, we could not record spontaneous speech of subjects because of its difficulty to conduct recording spontaneous speech without a facilitator. Instead, we could control the contents of speech between the subjects. We think that comparison between read speech and spontaneous speech is a future issue.

This study was a pilot study to examine feasibility therefore the number of the subjects is small. We are planning to conduct a further experiment with larger population.

## Conclusion

In this study, emotion analysis technology based on voices was applied in an effort to conduct a regular analysis of the mental homeostasis of a person making utterances.

A comparison of the values obtained in the overall sessions for different groups of test subjects clarified that there were tendencies with effect sizes ranging from 0.1477 to 0.3066 but not significant differences between the VAD-L, VAD-H, and VAD-R indices, calculated using the algorithms proposed by this paper, of two groups that were subjected for differing durations to an environment presumed to be stressful.

Although it was hypothesized that short and long durations in disaster zones would result in different VAD values due to potential mental health differences, the mental health of participants was not assessed by psychiatrists or using gold standard methods such as the General Health Questionnaire 30-item version [15]. Ascertaining correlations between VAD indices and established mental health assessments would provide a more comprehensive validation of voice emotion analysis such as that using ST software applied to vocal behavior.

Because the acquisition of voice recordings is less invasive and places less burden on test subjects than obtaining the typical physical information used for medical purposes, taking blood samples for example, VAD promises to be a useful approach for everyday use in screening and long-term continuous monitoring. In order to implement this method as a filtering tool for medically discovering depressed patients or manic patients, it will be beneficial to establish its sensitivity and degree of uniqueness to the mental illnesses of depressive and manic states; this will necessitate the study of detailed evaluation standards, including those of medical specialists, concerning states that need to be distinguished in test subjects.

## Abbreviations

ST: Sensibility Technology
VAD: vocal affect display
